# Changes in the nuclear proteome of developing wheat (*Triticum aestivum* L.) grain

**DOI:** 10.3389/fpls.2015.00905

**Published:** 2015-10-28

**Authors:** Titouan Bonnot, Emmanuelle Bancel, Christophe Chambon, Julie Boudet, Gérard Branlard, Pierre Martre

**Affiliations:** ^1^UMR1095 Genetics, Diversity and Ecophysiology of Cereals, Institut National de la Recherche AgronomiqueClermont-Ferrand, France; ^2^UMR1095 Genetics, Diversity and Ecophysiology of Cereals, Blaise Pascal UniversityAubière, France; ^3^Metabolism Exploration Platform Proteomic Component, Institut National de la Recherche AgronomiqueSaint-Genès Champanelle, France

**Keywords:** wheat, developing grain, nuclear proteins, 2D gel electrophoresis, LC-MS/MS

## Abstract

Wheat grain end-use value is determined by complex molecular interactions that occur during grain development, including those in the cell nucleus. However, our knowledge of how the nuclear proteome changes during grain development is limited. Here, we analyzed nuclear proteins of developing wheat grains collected during the cellularization, effective grain-filling, and maturation phases of development, respectively. Nuclear proteins were extracted and separated by two-dimensional gel electrophoresis. Image analysis revealed 371 and 299 reproducible spots in gels with first dimension separation along pH 4–7 and pH 6–11 isoelectric gradients, respectively. The relative abundance of 464 (67%) protein spots changed during grain development. Abundance profiles of these proteins clustered in six groups associated with the major phases and phase transitions of grain development. Using nano liquid chromatography-tandem mass spectrometry to analyse 387 variant and non-variant protein spots, 114 different proteins were identified that were classified into 16 functional classes. We noted that some proteins involved in the regulation of transcription, like HMG1/2-like protein and histone deacetylase HDAC2, were most abundant before the phase transition from cellularization to grain-filling, suggesting that major transcriptional changes occur during this key developmental phase. The maturation period was characterized by high relative abundance of proteins involved in ribosome biogenesis. Data are available via ProteomeXchange with identifier PXD002999.

## Introduction

Wheat (*Triticum aestivum* L.) grain is a major staple crop in many parts of the world. The end-use value is determined by complex molecular interactions that occur during grain development. Development of wheat grain is typical of grass seeds and is commonly subdivided into three developmental phases that overlap (Sabelli and Larkins, 2009). After double fertilization, the triploid endosperm divides successively without cytokinesis leading at 70°Cd after anthesis (i.e., 3–4 days after anthesis at an average daily temperature of 20°C) to the formation of a coenocyte whose nuclei are distributed throughout the endosperm (Mares et al., 1975). Cellularization follows which is a phase of cell division and differentiation until 220°Cd (11 days) after anthesis (Chojecki et al., 1986). The effective grain-filling phase follows when storage compounds, mainly starch and proteins, rapidly accumulate (Shewry et al., 2012). The rate of accumulation of starch and proteins slows down at around 550°Cd (27 days) after anthesis, when endosperm nuclei and protein bodies become compressed by starch granules (Hoshikawa, 1962; Ferreira et al., 2012) and progressively disintegrate. Accumulation stops at 650–700°Cd (32–35 days) after anthesis when the concentration of water in grain is close to 45 g per 100 g of fresh mass (Schnyder and Baum, 1992). Grains then start a phase of rapid desiccation and maturation during which desiccation tolerance is acquired. Because of the importance of wheat grain in the human diet, much research has focused on identifying processes which regulate these different phases of development in order to optimize grain yield and its quality (Shewry et al., 2012).

The regulation of most of these processes involves transcriptional regulation and the nucleus plays a key role in the regulation of grain development and storage compound accumulation. In plants, the nuclear proteome of leaves or whole seedlings has been studied for several species (Erhardt et al., 2010; Petrovská et al., 2015) including cereals like *Oryza sativa* (Khan and Komatsu, 2004; Tan et al., 2007; Aki and Yanagisawa, 2009; Choudhary et al., 2009; Jaiswal et al., 2013), *Hordeum vulgare* (Petrovská et al., 2014), and *Zea mays* (Ferreira et al., 2006; Guo et al., 2014). However, there have been few such studies on seeds (Repetto et al., 2012). In *O. sativa*, 468 nuclear proteins were identified from endosperm at 9 days after pollination (dap) (Li et al., 2008) and in *Medicago truncatula* 143 different nuclear proteins were identified from whole seeds harvested at 12 dap (Repetto et al., 2008). A study of *Z. mays* showed that some nuclear proteins extracted from endosperm isolated from grains harvested between 8 and 35 dap, analyzed on one-dimensional (1D) gels, were more abundant at certain times of development (Ferreira et al., 2006), but these proteins remain to be identified. No proteomic study has analyzed the temporal changes in abundance of nuclear proteins during grain development. However, identifying and quantifying nuclear proteins is an important step in characterizing some of the numerous regulatory mechanisms that take place during the dynamic phases of grain development. We hypothesized that the developmental physiology and morphology of the wheat grain requires changes in abundance of several nuclear proteins at specific times of grain development.

The aim of the present study was to analyze the nuclear proteome of the developing wheat grain in order to obtain a first overview of which nuclear proteins vary in abundance during grain development. Nuclear proteins were extracted from wheat (*T. aestivum* L.) grains collected during the cellularization, effective grain-filling and maturation phases of development, and analyzed using two-dimensional (2D) gel electrophoresis and electrospray ionization ion trap mass spectrometry (ESI-IT-MS/MS). This allowed us to show that some nuclear proteins involved in signaling, proteolysis, transcription regulation or ribosome biogenesis were more abundant at specific developmental phases or phase transitions.

## Material and methods

### Plant material

Plants of hexaploid winter wheat (*T. aestivum* L.) cv Recital were used in this study. Seeds were sown in plug trays filled with a peat moss mixture and were kept in a greenhouse until the ligule of the third leaf appeared. Air temperatures in the greenhouse were maintained at 18/10°C (light/dark) and air relative humidity at 70/50% (light/dark). Plants were then vernalized for 8 weeks in a growth chamber where the air temperature was maintained at 4 ± 1°C, the air relative humidity at 40% and the mean daily photosynthetic photon flux density (PPFD) at the top of the plants at 43 mmol m^−2^ d^−1^ during the 8-h photoperiod. After vernalization, the plants were transplanted into 293-mL plastic pots filled with a mixture of soil-pozzolan (2:1, w/w) and transferred to a walk-in growth chamber. The conditions in the growth chamber were 20/15°C (light/dark), 55/75% air relative humidity (light/dark), with an average PPFD of 550 μmol m^−2^ s^−1^ at top of the plants during the 16-h photoperiod. Plants were irrigated twice a day with a commercial nutrient solution.

Air temperature at the top of the plants was measured continuously. Main stems were tagged when the anthers of the central florets emerged (anthesis date). The sum of mean daily air temperature after anthesis was calculated to follow grain development in thermal time in degree-days (°Cd) above 0°C after anthesis. Grains were harvested at 150, 250, 350, 450, 600, and 750°Cd after anthesis and stored at −80°C. Only grains of the first floret from the central part of the ears were collected (approximately 10 grains per ear). Four independent replicates were used.

### Nuclei isolation and nuclear protein extraction from wheat grains

The method used in the present study to purify nuclei from wheat grains and to extract nuclear proteins was recently validated by Bancel et al. (2015). The authors verified the absence of contamination from non-nuclear proteins by western blots with primary antibodies which detect protein markers of the different sub-cellular compartments.

Briefly, nuclei were isolated from 2 g of grains. Grains were ground in extraction buffer [20 mM Hepes-KOH, pH 7, 5 mM MgCl_2_, 10 mM 2-ME, 0.5 mM PMSF, 0.1% (v/v) phosphatase inhibitor cocktail (Sigma-Aldrich)] with a Polytron homogenizer (Kinematica POLYTRON® PT 10) during 1 min. The homogenate was filtered through two layers of Miracloth (Calbiochem) to remove cell debris and cells were lysed by adding 0.5% (v/v) Triton X-100. After incubation for 15 min at 4°C, the resulting lysate was centrifuged at 1000 × *g* for 10 min at 4°C. Each pellet was then washed four times by resuspension in 1 mL of extraction buffer followed by centrifugation at 1000 × *g* for 10 min at 4°C. Nuclei were purified from the pellet by centrifugation at 930 × *g* for 30 min at 4°C through a stepwise Percoll density gradient, 30–80% Percoll prepared in extraction buffer. Nuclei floating at the interface were collected and washed twice with 3 mL of extraction buffer followed by centrifugation at 3500 × *g* for 5 min at 4°C. To verify the purity of isolated nuclei, nuclei pellets were washed in 500 μL of PBS (137 mM NaCl, 2.7 mM KCl, 4.3 mM Na_2_HPO_4_, 1.47 mM KH_2_PO_4_, pH 7.4) and centrifuged at 3500 × *g* for 5 min at 4°C. The supernatant was removed and nuclei were stained in phosphate buffered saline (PBS) solution containing 0.1 μg/mL Hoechst for 5 min in the dark. After two washes in 50 μL of PBS, 5 μL aliquots were observed under fluorescence microscopy (Zeiss Axioplan 2 microscope). To verify absence of pigments in nuclei pellets, a chlorophyll assay was performed according to (Pandey et al., 2006). Briefly, 100 μL of sample was mixed with 100 μL of water and 800 μL of acetone. After centrifugation at 1000 × *g* for 5 min, the optical density was measured at 652 nm. The amount of chlorophyll was observed as μg per μL by calculating optical density/34.5 (at 652 nm chlorophyll a and b intersect, 34.5 is the specific absorption coefficient for both pigments at this wavelength).

Nuclear proteins were prepared using TRI Reagent® (Sigma-Aldrich) according to the manufacturer's instructions. The final protein pellet was dried under ambient conditions for 1 h and then stored at −20°C.

### SDS-PAGE analysis and immunoblotting using anti-histone H3 antibody

Nuclear protein pellets and proteins from supernatants (S1–S5) collected during nuclei purification were solubilized in 100 μL of solubilization buffer (45 mM Tris-HCl, pH 6.8, 50 mM DTT, 1% (w/v) SDS, 10% (v/v) glycerol, 0.001% (w/v) bromophenol blue). A fixed volume (35 μL of each supernatant or 25 μL of final nuclear protein extract) was loaded onto 12.5% SDS-polyacrylamide gels as described in (Bancel et al., 2015). For immunoblotting analysis, proteins were transferred from the 1D gels to nitrocellulose membranes (Hybond ECL, GE Healthcare) during 1 h in a semidry unit apparatus (GE Healthcare). Membranes were incubated with an anti-histone H3 antibody (Abcam) diluted at 1:1000. Membranes were then incubated with anti-rabbit secondary antibody coupled to horseradish peroxidase (HRP, GE Healthcare) diluted at 1:5000. The chemiluminescence was developed according to the manufacturer's instructions (ECL Western Blotting, SuperSignal West Pico Chemiluminescent Substrate kit, Amersham).

### Two-dimensional electrophoresis of nuclear proteins

Dried pellets containing nuclear proteins were dissolved in 50 μL of 2D gel sample buffer [7 M urea, 2 M thiourea, 4% (w/v) CHAPS, 70 mM dithiothreitol, 1% (v/v) immobilized pH gradient (IPG) buffer (either for the pH 4–7 range or the pH 6–11 range), and 0.34% (v/v) protease inhibitor (Sigma-Aldrich)] for 1 h at room temperature with constant agitation. An aliquot (5 μL) of each sample was used to quantify protein content (Bradford, 1976) using bovine serum albumin as standard. Isoelectric focusing was carried out with 150 μg of proteins, made up to 250 μL with the 2D buffer containing 0.05% (w/v) bromophenol blue and used to passively rehydrate 13-cm immobilized pH gradient strips (pH range 4–7 or 6–11 Immobilin Dry Strips, GE Healthcare) overnight at 20°C. For the comigration gels, made to be a reference for the digital analysis step, isoelectric focusing was performed with 240 μg of proteins (40 μg of nuclear proteins from each of the six thermal times after anthesis). Isoelectric focusing was performed for a total of 60,000 voltage hours (Vhr) on a IPGphor II apparatus (GE Healthcare). Focused proteins on strips were then reduced with 2% (w/v) dithiothreitol in 0.1 M Tris-HCl buffer (pH 8.8) containing 6 M urea, 30% (v/v) glycerol, and 2% (w/v) SDS for 15 min, followed by alkylation with 2.5% (w/v) iodoacetamide in the same buffer for 15 min. The strips were then loaded onto 12.5% polyacrylamide gels for SDS-PAGE separation in the second dimension. The migration conditions were 10 mA per gel for the first 30 min, then 35 mA per gel for 2.5 h. Gels were stained using Coomassie Brilliant Blue G250 (CBB, Sigma-Aldrich) (Neuhoff et al., 1985). To improve detection of low abundance protein spots and allow their collection, gels were destained overnight in a solution containing 40% (v/v) ethanol and 10% (v/v) acetic acid and silver-stained following a mass spectrometry compatible method (Shevchenko et al., 1996).

### Image and statistical analyses

Images (300 dpi, 16-bit greyscale pixel depth) of two-dimensional gels stained with CBB were acquired with a GS-800 (Biorad) scanner and analyzed using SameSpots v4.5 (TotalLab) 2D gel image analysis software. Statistical analyses were performed on normalized protein spot volume values. Differences in normalized protein spot volume due to grain development were analyzed using One-way ANOVA. *P*-values and adjusted *P*-values (*q*-value) were calculated using SameSpots procedures. The abundance of a protein spot was considered to have changed during grain development when its *P*-value and *q*-value were both < 0.05. In this case, the protein spot was considered as “variant” and others as “non variant.” Principal component analysis was performed using the FactoMineR (Husson et al., 2014) package for R v3.0.1 (R Core Team, 2013) statistical software on the set of spots detected on 2D gels and hierarchical clustering on principal components was computed on significant spots to build protein abundance profiles.

### Protein identification

Protein spots were excised manually from 2D gels. For 2D gels stained with CBB, protein spots were destained once with 25 mM NH_4_HCO_3_ containing 5% (v/v) acetonitrile (ACN) for 30 min and twice with 25 mM NH_4_HCO_3_, 50% (v/v) ACN for 30 min. Spots were then dehydrated in 100% ACN for 10 min and dried for 15 min under an extraction hood at room temperature. For 2D gels stained with silver nitrate, protein spots were first destained with 30 mM K_3_Fe(CN)_6_, 100 mM Na_2_S_2_O_3_ for 1–2 min, then washed twice in water for 15 min, before following the CBB destaining steps as above. Proteins were digested overnight at 37°C by adding 120 ng of trypsin (Promega). After extracting peptides with ACN, 8 μL of hydrolysate were injected into an Ultimate® 3000 HPLC system (Dionex) coupled to an electrospray ionization ion trap mass spectrometer (ESI-IT-MS/MS; LTQ Velos, ThermoScientific).

Protein identity was sought by using Mascot v2.3 (Matrix Science) software against a custom database containing 249,032 sequences from *Aegilops tauschii, A. thaliana, Brachypodium distachyon, H. vulgare, O. sativa, T. aestivum*, and *T. urartu* and sequences from the wheat transcription factor database wDBFT (Romeuf et al., 2010). Proteins were considered to be identified if at least two non-redundant peptides were found to match a single reference in the databases. A cut-off was applied for individual peptide ion scores according to the significance threshold of the MASCOT program (*P* < 0.05). Curated protein sequences which had no functional information were submitted as BLASTP searches against the National Center for Biotechnology Information non-redundant database (http://blast.ncbi.nlm.nih.gov). Proteins were then classified in functional classes according to the KEGG PATHWAY database (Kanehisa et al., 2014) and gene ontology (Ashburner et al., 2000). Subcellular localization of identified proteins was predicted by *in silico* analysis using Multiloc2 (Blum et al., 2009), WolfPSort (Horton et al., 2007), Y loc (Briesemeister et al., 2010), and LocTree (Goldberg et al., 2014) programs. The top two hits were considered for Multiloc and Y loc. The mass spectrometry proteomics data have been deposited to the ProteomeXchange Consortium (Vizcaíno et al., 2014) via the PRIDE partner repository with the dataset identifier PXD002999.

## Results

### Nuclei purified and nuclear proteins extracted from developing wheat grains

**Figure 1:**
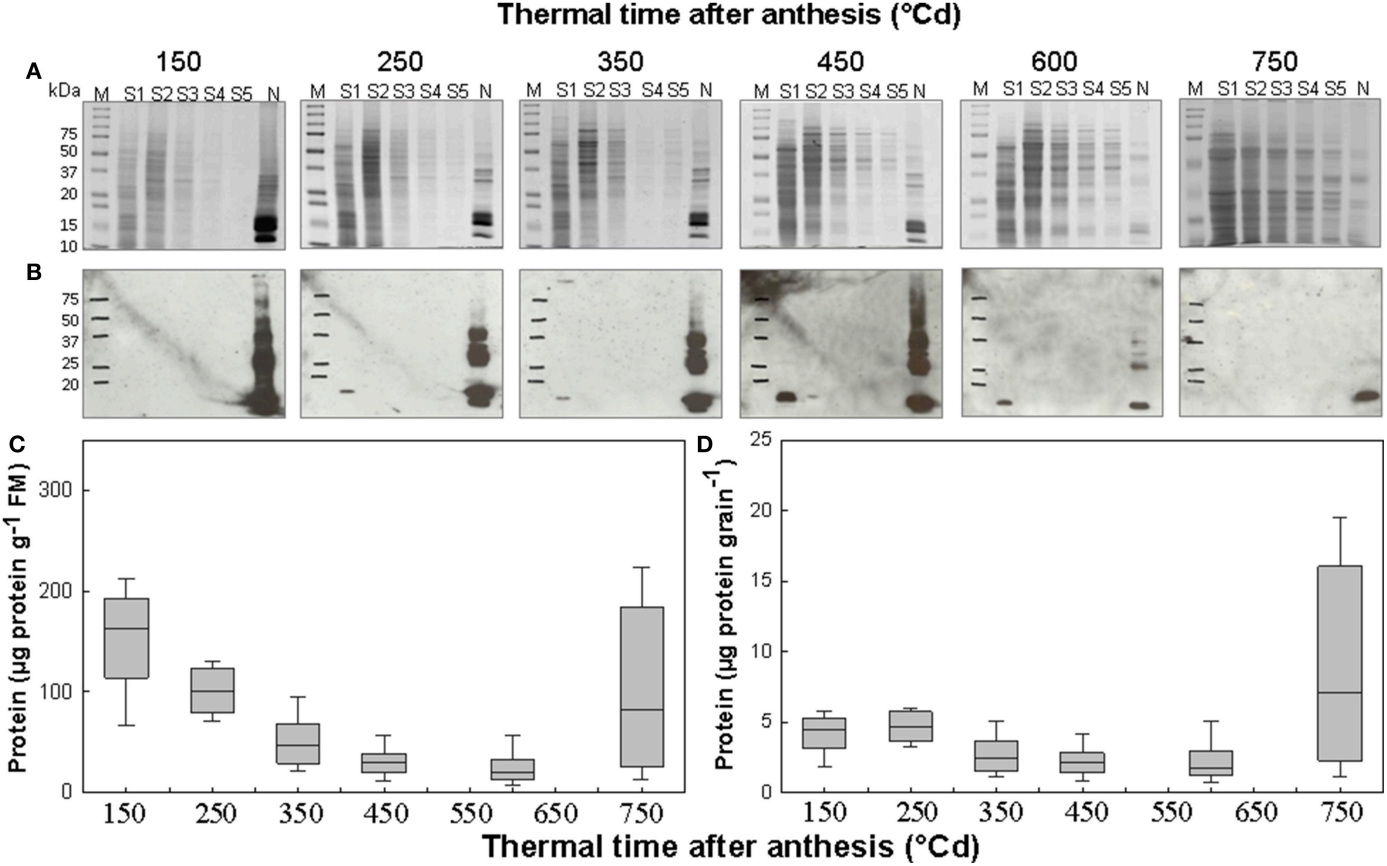
**Nuclear proteins extracted from developing wheat grains**. Proteins from supernatants (S1–S5) and nuclear-enriched fractions (N) for each thermal time after anthesis (150–750°Cd) were analyzed on SDS-PAGE **(A)** then western blot analysis was performed using antibody directed against histone H3 **(B)**. M, molecular weight protein standards. Amount of proteins in the nuclear-enriched fraction per fresh mass (FM) of grain **(C)** or per grain **(D)** vs. thermal time after anthesis. In **(C,D)** boxes show the 25th to 75th percentile range, horizontal lines in boxes show medians, and error bars outside boxes show the 10th to 90th percentile range for *n* ≥ 10 independent extractions.

Here we analyzed the nuclear proteome of developing wheat grain. For this, grain was harvested at six thermal times corresponding to the cellularization phase (150°Cd after anthesis), at the transition between cellularization and grain-filling phases (250°Cd after anthesis), during the grain-filling phase (350 and 450°Cd after anthesis), at the transition between grain-filling and maturation phases (600°Cd after anthesis), and during the maturation phase (750°Cd after anthesis). Nuclei were isolated from wheat grains using Percoll density gradient purification. The integrity of isolated nuclei was verified by Hoechst staining. For all stages of grain development studied, Hoechst staining showed that nuclei with an average diameter of approximately 20 μm had been purified (Supplementary Figure 1A). Chlorophyll assay was also performed which showed that nuclear extracts were free of these pigments (Supplementary Figure 1B). Nuclear proteins and proteins from supernatants collected during nuclei purification were analyzed by SDS-PAGE and western blot analysis with an anti-histone H3 antibody (Figures 1A,B). For each stage of grain development, 1D protein profiles of the nuclear fractions were different from those of supernatants (Figure 1A). After blotting, histone H3 proteins were clearly detected in the nuclear fraction, confirming that the protein extracts were enriched in nuclear proteins (Figure 1B). Histone H3 was less abundant in supernatant S1 from protein samples from 250, 350, 450, and 600°Cd after anthesis indicating that a few nuclei were lost during purification.

The 1D gels show that the quantity of nuclear proteins extracted from an equal mass of grain decreased during grain development. This result was confirmed by protein assays of the nuclear protein fraction (Figure 1C). However, the amount per grain of nuclear proteins extracted at various thermal times after anthesis were similar, except at 750°Cd after anthesis when the variability was too high to evaluate this (Figure 1D).

### Two-dimensional electrophoresis of wheat grain nuclear proteins

**Figure 2:**
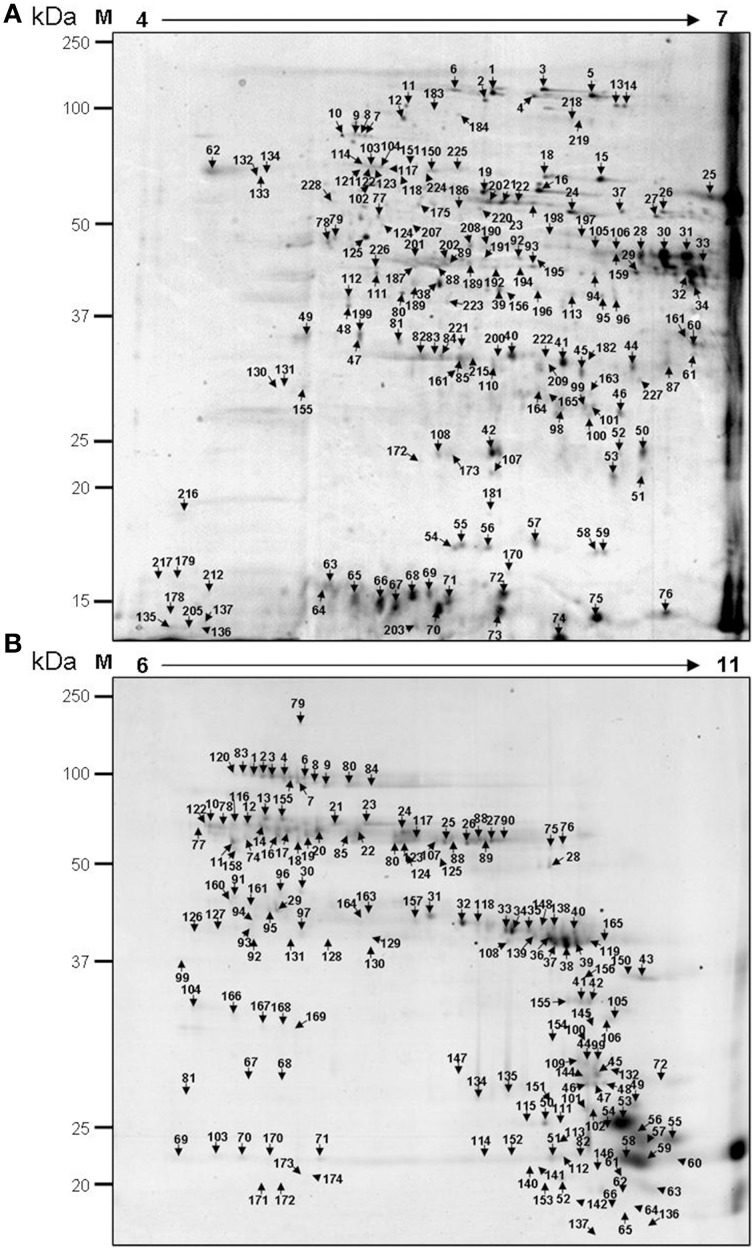
**Map of wheat grain nuclear protein spots**. Comigration in 2-D electrophoresis of nuclear proteins extracted from grains harvested at six thermal time after anthesis. Proteins were loaded onto 13-cm IPG strips in the pH range 4–7 **(A)** and 6–11 **(B)** for isoelectric focusing in the first dimension and SDS-PAGE in the second dimension. M, molecular weight protein standards. Protein spots that were excised and analyzed by mass spectrometry are indicated.

To maximize resolution of protein spots in 2D electrophoresis, two different pH gradients were used in the first dimension of isoelectric focusing. Comigration gels are shown in Figure 2 in which samples from all 6 stages of wheat grain development were combined. With the pH 4–7 gradient, 371 spots were detected and with the pH 6–11 gradient 299 spots were detected. From this total of 690 protein spots, 213 were manually excised from the pH 4–7 gradient gel and 174 from the pH 6–11 gradient gel, without any a priori (Figure 2). From the total of 387 excised spots, 343 (88.6%) polypeptides were identified by LC-ESI-MS/MS, which correspond to 114 different proteins (Supplementary Tables S1, S2). In many cases, the same protein was found in multiple spots, indicative of isoforms and/or post-translational modifications.

**Figure 3:**
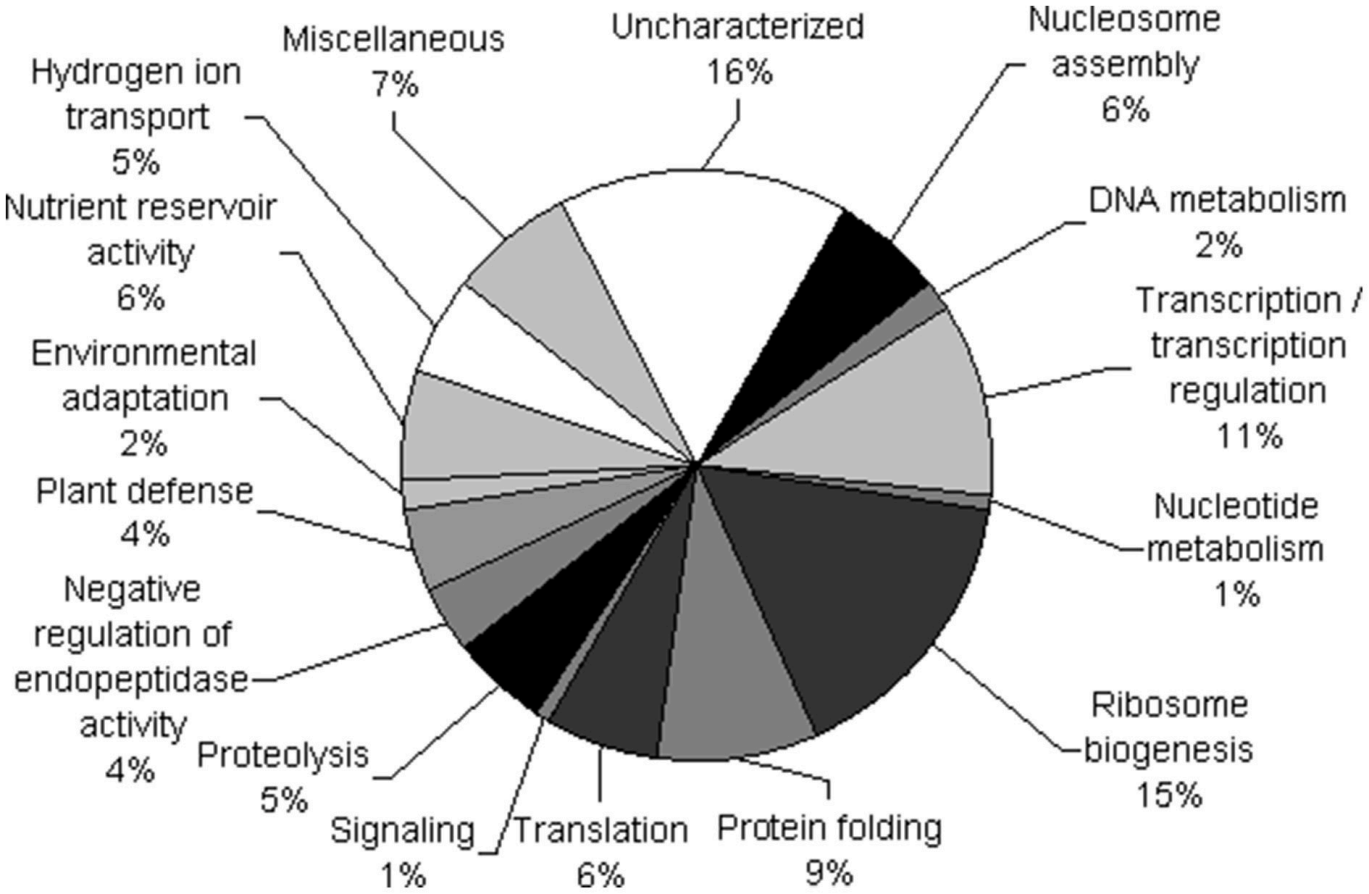
**Functional classification of nuclear proteins**. The 114 different proteins identified were grouped into 16 functional classes according to the KEGG pathway database and gene ontology (GO). The percentage of proteins in each functional class is shown.

The identified proteins were organized into 16 functional classes (Figure 3 and Supplementary Table S1). Among the proteins with known functions, a large proportion (15%) corresponded to ribosomal proteins involved in ribosome biogenesis. The second largest category (11%) comprised proteins involved in transcription and transcription regulation. For example, DNA-directed RNA polymerases I, II, and III subunit rpabc3 is involved in RNA synthesis, RNA helicases, arginine/serine-rich splicing factor, and Mago nashi-like protein are all involved in mRNA processing. Transcriptional regulators were also identified such as a MADS-box transcription factor and a HMG1/2-like protein. Three spots corresponded to histone deacetylases, which mediate the deacetylation of lysine residues on the N-terminal part of core histones and three others matched the FACT complex subunit SSRP1-B which regulates transcription by modifying nucleosomal structure. In the same category, WD repeat-containing protein RBAP1 was also identified. Proteins involved in protein folding represented the third largest category (9%) among characterized proteins. Other proteins were related to translation (6%) including initiation and elongation factors. In the nucleosome assembly category (6%) histones H1, H2A, H2B, and H4 were identified. Finally, some proteins with storage functions were identified in the nutrient reservoir activity category (6%) and are thought to be contaminants from the purification method. The function of 17 (16%) proteins was not known so they were classified as uncharacterized.

### The relative abundance of many nuclear proteins varied during grain development

For the six thermal times after anthesis, CBB stained gels from four biological replicates were analyzed by digital imaging. Principal component analysis was performed with the normalized volumes of the 690 protein spots detected by image analysis (Section Two-dimensional Electrophoresis of Wheat Grain Nuclear Proteins). The four replicates for a given time point segregated away from those of other time points (Supplementary Figure 2). From the 690 protein spots detected, 226 (33%) had a constant relative abundance during grain development, and 464 varied significantly (67%). Protein spots were excised without any a priori. In this way, among the 387 protein spots analyzed by LC-MS/MS, 153 (40%), corresponding to 69 different proteins, did not vary in relative abundance during grain development, and are qualified as non-variant, whereas 234 (60%), corresponding to 72 different proteins, were variant. An interesting initial conclusion is that a protein can be identified in multiple spots, some of which vary during grain development, and others which do not.

**Figure 4:**
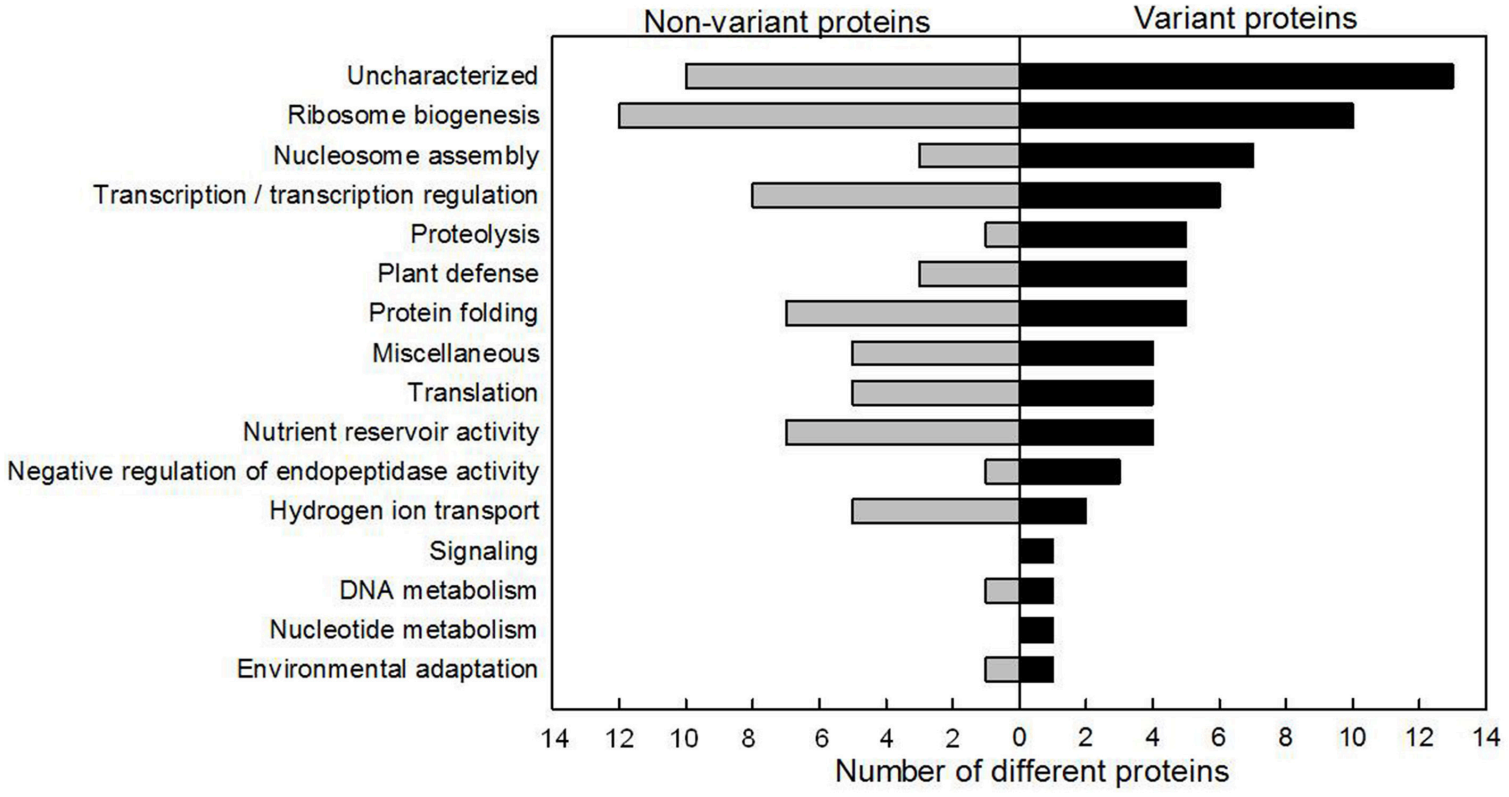
**Functional distribution of non-variant and variant nuclear proteins**. Proteins identified showing no statistically significant change are represented on the left and the variant proteins are presented on the right.

Proteins involved in functional classes ribosome biogenesis (12 proteins), uncharacterized (9 proteins) and transcription/transcription regulation (8 proteins) were the most numerous among the non-variant proteins (Figure 4). The ribosome biogenesis functional class (10 proteins) was also highly represented among the variant proteins (Figure 4). The relative abundance of seven histones varied significantly. Only 50% (6/12) of the identified proteins related to transcription regulation varied during grain development. Conversely, the functional classes of proteolysis and plant defense were more highly represented in the variant protein group (five different proteins from each class) than in the non-variant protein group (1 and 3 different proteins in each class, respectively).

**Figure 5:**
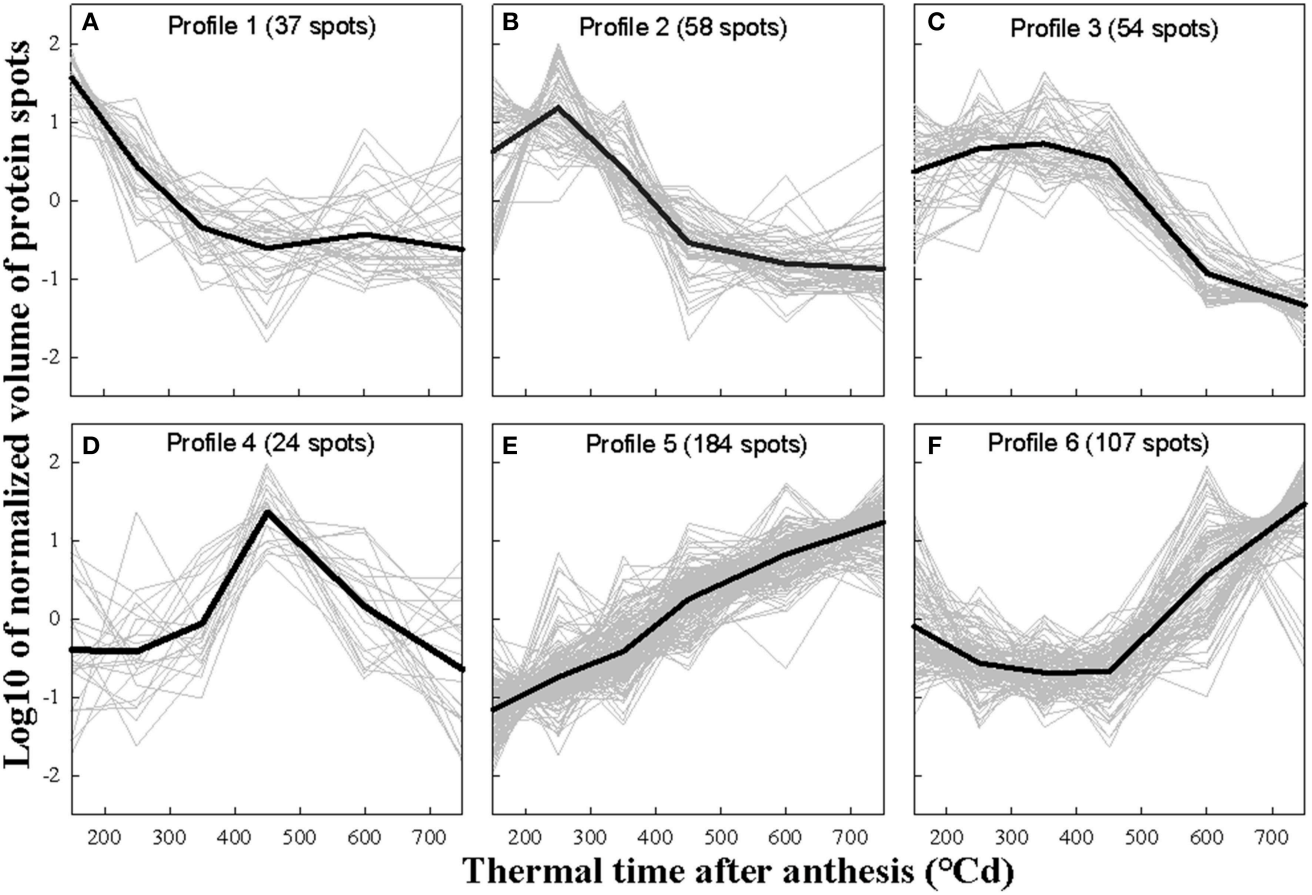
**Abundance profiles of variant protein spots**. Six profiles **(A–F)** were established from the normalized volumes of the 464 variant spots using hierarchical clustering on principal components. Data are means of *n* = 4 replicates. In each profile, the black line represents the mean. The number of protein spots in each profile is given in parenthesis after the profile number.

The clustering analysis was first performed separately on the pH gradients. Since the two pH ranges gave similar clusters, the clustering analysis presented here was performed using data from the two pH ranges. The 464 variant protein spots detected by image analysis were grouped into six profiles according to their relative abundance at different stages of grain development (Figure 5). Profile 1 included 37 spots with a maximum normalized volume at 150°Cd after anthesis that decreased to a minimum value at 450°Cd after anthesis (Figure 5A). Profile 2 grouped 58 spots whose normalized volume peaked at 250°Cd after anthesis (Figure 5B). The 54 spots that defined profile 3 had a maximum normalized volume between 150 and 450°Cd after anthesis (Figure 5C). Profile 4 grouped 24 spots with normalized volume that peaked at 450°Cd after anthesis (Figure 5D). Profile 5 grouped 184 spots whose normalized volume increased throughout grain development (Figure 5E) and profile 6 included 107 spots whose normalized volume decreased from 150 to 450°Cd after anthesis and then increased until 750°Cd after anthesis (Figure 5F).

Each of the 72 identified variant proteins had at least one of the six profiles (Supplementary Table S1). There were 12 proteins with profile 1, 19 with profile 2, 15 with profile 3, 6 with profile 4, 21 with profile 5, and 21 with profile 6. In some cases therefore the same protein, perhaps different isoforms, had different abundance profiles. Profiles 1 and 2 were characterized by proteins involved in transcription and transcription regulation. For example, HMG1/2-like protein, Mago nashi-like protein and a histone deacetylase HDAC2 were more abundant during the cellularization phase of grain development (profile 1). Two helicase proteins and a HMG1/2-like protein peaked in relative abundance at the end of the cellularization phase (profile 2). Seven histones had profile 3 indicating that they were more abundant during the cellularization phase and the beginning of the grain-filling phase. Several variant ribosomal proteins had profiles 4, 5 and 6, and therefore were more abundant as a class during the latter part of the grain filling phase and during maturation. Proteins with profile 6, increasing steadily in abundance during the late grain-filling phase, included three serpins, which are known to negatively regulate endopeptidase activity, four proteins involved in protein folding and two proteins involved in plant defense.

### Focus to variation in abundance for some identified protein

**Figure 6:**
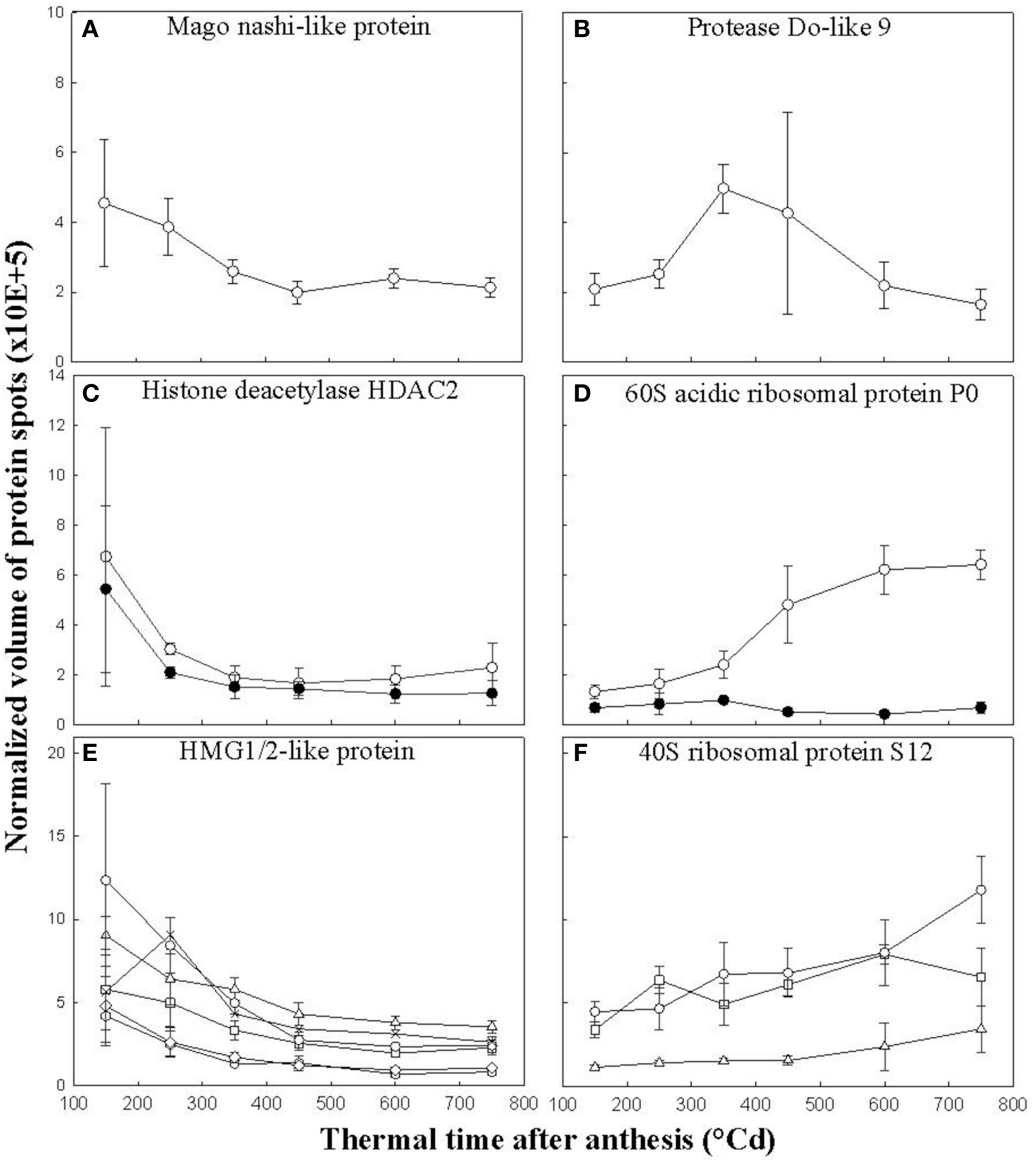
**Changes in volumes of several nuclear protein spots**. Mago nashi-like protein **(A)** and protease Do-like 9 **(B)** were each identified in only one spot whose abundance varied significantly during grain development. Histone deacetylase HDAC2 **(C)** and 60S acidic ribosomal protein P0 **(D)** were each found in two spots including one which varied significantly (open circles) and one which did not vary significantly during grain development (closed circles). HMG1/2-like protein was identified in eight spots including six that varied, presented graphically in **(E)**. The 40S ribosomal protein S12 **(F)** was found in three spots whose volumes varied significantly during grain development. Data are means ± 1 s.d. for *n* = 4 independent replicates.

In some cases, only one spot matched a unique protein and the volume of this spot varied significantly during seed development, e.g., the Mago nashi-like protein and the Do-like 9 protease with profile 1 (Figures 6A,B). In 25% of the cases (28 different proteins), several protein spots corresponding to the same protein did not vary significantly in the same way. For example, two protein spots corresponded to a histone deacetylase HDAC2. One spot varied as in profile 1, but the other did not vary during grain development (Figure 6C). This was also the case for a 60S acidic ribosomal protein P0 protein. One protein spot peaked in relative abundance at 600°Cd after anthesis, while the other spot remained constant (Figure 6D). In a few cases (16%), several protein spots corresponding to the same protein were variant but did not have the same relative abundance profile. For example, an HMG1/2-like protein was present in eight protein spots. Of these, two varied with profile 1 dynamics and four with profile 2 dynamics (Figure 6E). Nevertheless overall this protein was more abundant during the cellularization phase. Finally in some cases, a protein was identified in several spots which all showed the same abundance dynamics. For example, 40S ribosomal protein S12 was identified in three spots, which were all classified as profile 5 (Figure 6F), all increasing in abundance during grain development.

## Discussion

DNA replication, repair and modification, RNA transcription, and ribosome biogenesis occur in the nucleus. Continuous flux of RNAs and proteins across the nuclear envelope make the nucleus a very dynamic structure and the site of developmental regulation. We analyzed the nuclear proteins that were extracted from nuclei at key stages of wheat grain development. Using 2D electrophoresis and LC-ESI-MS/MS, 114 different proteins were identified. Changes in the relative abundance of proteins and their isoforms during grain development are highlighted. Certain proteins which could have a potential role in the regulation of grain development are discussed, and could indeed be the object of further studies.

### Nuclear proteins identified provide information on the functions of wheat grain nuclei

After translation in the cytoplasm, ribosomal proteins are transported to the nucleolus, where ribosome assembly begins (Fromont-Racine et al., 2003; Boisvert et al., 2007). Among the 114 identified proteins, 18 (15%) are ribosomal proteins. By comparison, in a study of the *M. truncatula* nuclear proteome, 32 ribosomal proteins (22%) were identified (Repetto et al., 2008). The second largest category of proteins (12 proteins) identified were those involved in transcription/transcription regulation. Some of them have been identified in previous studies of grain nuclear proteomes, such as the histone deacetylases (Li et al., 2008; Repetto et al., 2008). However, the HMG1/2-like protein, identified here in eight protein spots, had not previously been found in the nuclear proteome of grain from other species. The presence of histone proteins was expected as they are involved in nucleosome assembly, the first level of DNA compaction, and seven were indeed identified.

The presence of other proteins in the nucleus was harder to predict. Four different tools were used to predict subcellular localization. Seventy one percent of identified protein spots were predicted to correspond to a nuclear protein with at least two tools (N2, Supplementary Table S1), which suggest that these proteins correspond to nuclear actors or spend some time in the nucleus. However, proteins not predicted to be nuclear can't be excluded due to the limitation of the prediction tools. Indeed, several proteins known to spend some time in the nucleus were not predicted to be nuclear with the tools employed here, like for example some ribosomal proteins, involved in the ribosome biogenesis that occur in the nucleolus. While it is probable that some of proteins identified are contaminants from the purification process, it has been estimated that 35% of total proteins have multiple subcellular locations (Zhang et al., 2008), so some are worth discussing. For example, luminal binding proteins and HSP70 act mainly in the endoplasmic reticulum or in the cytosol to contribute to the formation of three-dimensional structures of proteins or protein complexes. These two types of protein were found in wheat grain nuclei. HSP70 has also been identified in the nucleus of other plant species (Calikowski et al., 2003; Pandey et al., 2006; Repetto et al., 2008) and some HSP are translocated to the nuclei of hamster fibroblasts following heat stress (Nollen et al., 2001). Two guanine nucleotide binding proteins were identified. They are commonly associated with the plasmalemma acting in multiple signal transduction pathways. In eukaryotes, guanine nucleotide binding proteins can also be associated with endomembranes, nucleus and the cytoskeleton (Willard and Crouch, 2000). Four serpin proteins were identified in this study. In mammals, several members of the serpin family have been found to localize in the nucleus and some have a nuclear localization signal (Silverman et al., 2001).

Translation mostly takes place in the cytosol. However, initiation and elongation factors involved in translation have been identified in the nuclear proteomes of *M. truncatula, O. sativa*, and here in *T. aestivum* (Repetto et al., 2008; Choudhary et al., 2009). Several studies have raised the possibility of nuclear translation (Dahlberg et al., 2003), which could take place in the nucleoplasm and the nucleolus in mammalian cells (David et al., 2012). Indeed the idea of a translasome has been described, a super-complex identified in the nucleus of yeast cells which would consist of an assembly of ribosomal proteins, elongation factors, proteasome, chaperones and tRNA synthetases (Sha et al., 2009).

Proteins classified in the nutrient storage activity group were identified in many protein spots. They are very abundant at the end of the grain-filling phase and probably are contaminants. Their presence may even explain the variability in the amount of proteins extracted from grains harvested at 750°Cd after anthesis.

### Constant need for ribosome biogenesis proteins increasing at the end of grain development

Among the 18 ribosomal proteins identified, six were present in multiple spots and for 10 at least one of their protein spots varied in relative abundance during grain development. These variant proteins had abundance profiles 3, 4, 5, or 6. Thus, ribosome formation seems to occur in each phase of grain development. However, more different proteins were present in profiles 4, 5, and 6 (nine different proteins in total) than in the other three profiles (one protein) and were thus more abundant as a class after 450°Cd after anthesis. Possibly there is a high demand for ribosome synthesis during the second half of the effective grain-filling and early maturation phases. This is somewhat similar to *M. truncatula* seed in which transcripts encoding genes involved in ribosome biogenesis were more abundant at the end of seed development (Repetto et al., 2008). This result suggests that there may be a ribosome pool formed at the end of the wheat grain development, which is necessary for grain germination. This is in accordance with results in *Arabidopsis thaliana* which showed that potential for seed germination is largely programmed during the seed maturation phase (Rajjou et al., 2004).

### Potential regulators of the beginning and end of the filling phase

RNA helicases 2 and 34 and Mago nashi-like proteins, all involved in mRNA maturation, were most abundant at 250 and 150°Cd after anthesis, respectively. Similarly, HMG1/2-like protein was most abundant during the cellularization phase. HMG are abundant DNA–binding chromosomal non-histone proteins which are not essential for chromatin organization but act with transcription factors in transcriptional control (Calogero et al., 1999; Jerzmanowski et al., 2000). They are probably also architectural factors in the assembly of certain nucleoprotein complexes (Jerzmanowski et al., 2000). Staining of the 2D gels with Pro-Q Diamond® (Invitrogen) revealed that one spot of this protein was phosphorylated at 150°Cd (Supplementary Figure 3). The histone deacetylase HDAC2 was most abundant at 150°Cd after anthesis. In *A. thaliana* this protein is one of 18 histone deacetylases involved in the repression of gene expression in multiple developmental processes by causing chromatin compaction (Hollender and Liu, 2008; Liu et al., 2014). Staining of the 2DE gels with Pro-Q Diamond® showed that the two spots corresponding to this protein were phosphorylated at 150°Cd after anthesis and one was still phosphorylated at 250°Cd after anthesis (Supplementary Figure 3). In mammals, many HDACs were found to be phosphorylated both *in vitro* and *in vivo* (Sengupta and Seto, 2004). This post-translational modification could affect the activity of this protein. These results suggest that some changes in transcriptional regulation occur at the transition between the cellularization and the grain-filling phases and that a number of genes are repressed in early grain development. In barley grain, a massive transcriptional reprogramming occurs during this developmental transition (Sreenivasulu et al., 2004, 2010) and the proteins identified in the present study may be potential regulators of this key stage.

Identified histones had a higher relative abundance between 150 and 450°Cd after anthesis than later during grain development and more variant types were also detected during this period. This was surprising as some histones have previously been shown to remain constant throughout wheat grain development with the synthesis of histones at 3 dap ending around 16 dap, approximately 320°Cd after anthesis (Spiker et al., 1987).

Several proteins accumulated in the nucleus at the end of grain development. For example, arginine/serine rich splicing factor which is known to be localized in nuclear specks and to be part of the spliceosome (Tillemans et al., 2006). Serpin proteins, which negatively regulate proteases, were also more abundant in late development, as previously observed in the endosperm of developing wheat grain (Tasleem-Tahir et al., 2012). Interestingly, proteins involved in proteolysis were more abundant between 150 and 450°Cd after anthesis, before serpins accumulate. Proteins with a role in mRNA maturation or in protection against degradation might regulate processes at the end of the grain-filling phase.

### Do non-variant nuclear proteins have a regulatory role during grain development?

Proteins which did not vary are likely to be essential throughout grain development and may still regulate grain development. An example is the FACT complex subunit SSRP1-B, which like HMG1-2 facilitates the formation of nucleoprotein structures (Röttgers et al., 2000). It may also act in protein complexes to control transcription mechanisms modulating the properties of chromatin. Another example is the histone deacetylase HDT2, which could repress transcription in the same manner as *Z. mays* HDAC2, by forming a complex of three polypeptides (Hollender and Liu, 2008). These proteins probably play an important role during grain development even though we didn't see any change in their abundance. Interestingly, the histone deacetylase HDT2 protein spot was stained with both CBB and Pro-Q Diamond® at 150 and 250°Cd after anthesis, indicating that this protein was phosphorylated at the end of the cellularization phase (Supplementary Figure 3). Perhaps post-translational modification such as phosphorylation regulates these proteins during grain development.

## Concluding remarks

**Figure 7:**
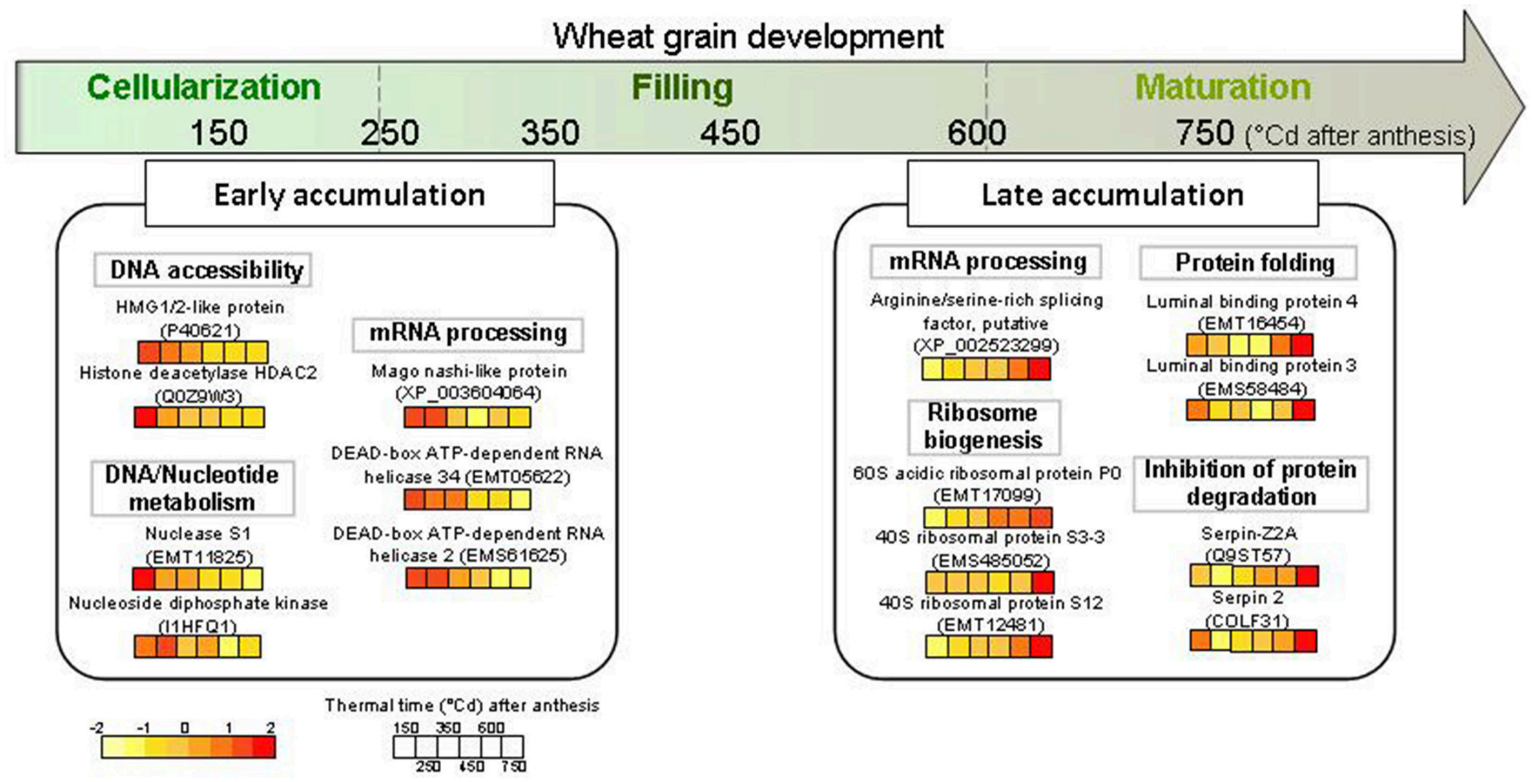
**Schematic view of some highlighted nuclear proteins accumulated during early and late grain development**. These proteins were extracted from wheat grain at six thermal times after anthesis, from 150 to 750°Cd and analyzed on 2-D gel electrophoresis. The color code for normalized volume of correspondent protein spots is indicated at the bottom left.

Some nuclear proteins are central actors in biological processes that regulate seed development. This study identified 114 different wheat proteins with various functions and dynamics, some of which had been found in previous studies of nuclear proteomes of other plant species and organs. For the first time, we have an overview of some of the quantitative changes occurring in 2D nuclear proteome of the developing wheat grain. This study revealed that the dynamics of the nuclear proteome of wheat grain seems to be divided into two periods (Figure 7). The first phase corresponds to the cellularization and early effective grain-filling phases, during which a change in transcription regulation occurs with a high abundance of proteins involved in mRNA processing. The second phase corresponds to the end of the effective grain-filling phase and the early maturation phase, when there is an activation of ribosome synthesis and an increase in proteins inhibiting protease action. This study opens the way for more precise research into the regulatory mechanisms that govern the accumulation of starch, storage proteins, and micronutrients that determine the processing and health value of cereal grains.

### Conflict of interest statement

The authors declare that the research was conducted in the absence of any commercial or financial relationships that could be construed as a potential conflict of interest.
