# Supplementary material for: Changes in the nuclear proteome of developing wheat (Triticum aestivum L.) grain
Source: Front Plant Sci. 2015 Oct 28;6:905. doi: 10.3389/fpls.2015.00905 (PMC4623401; doi:10.3389/fpls.2015.00905)
Supplement: Supplementary file 1 [file Image1.PDF]

frontiers

Supplementary Material

Changes in the nuclear proteome of developing wheat (*Triticum aestivum* L.) grain

Titouan Bonnot[1,2], Emmanuelle Bancel[1,2,*], Christophe Chambon[3], Julie Boudet[1,2], Gérard Branlard[1,2], and Pierre Martre[1,2,†]

*** Correspondence:** Emmanuelle Bancel: emmanuelle.bancel@clermont.inra.fr

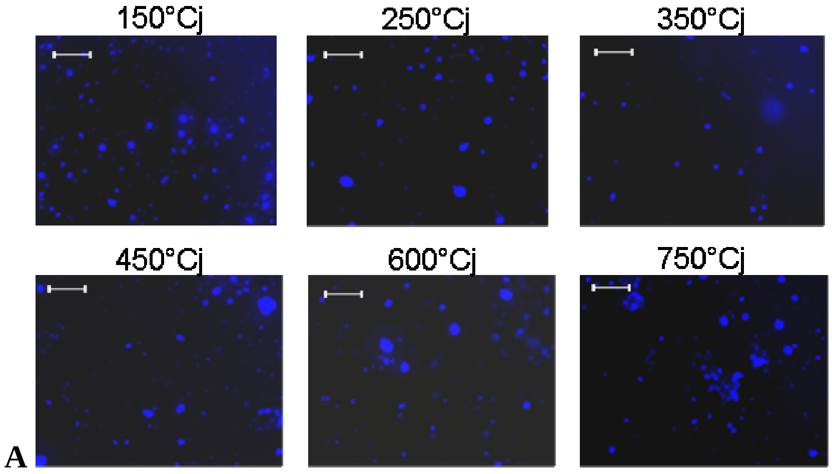

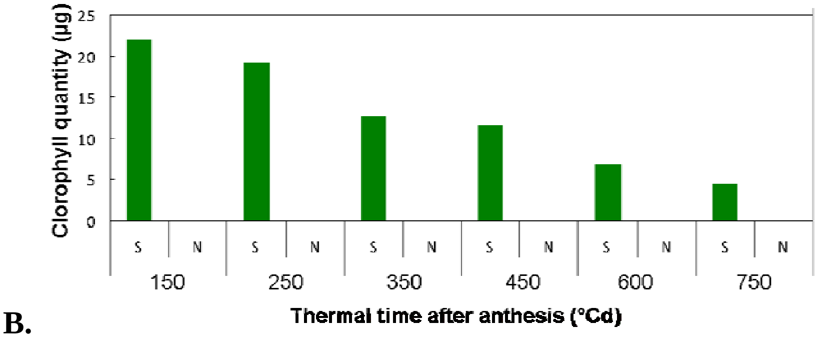

Supplementary Figure 1. Validation of nuclei purification. Nuclei were isolated from wheat grains harvested between 150°C and 750°Cd after anthesis. **A.** Nuclei were observed under fluorescence microscopy (Zeiss Axioplan 2 microscope). Scale bar represents 50 µm sizes.
B. Chlorophyll assay was performed on supernatants (S) collected during washing steps of nuclei and on nuclei extracts (N).
