# Supplementary material for: Changes in the nuclear proteome of developing wheat (Triticum aestivum L.) grain
Source: Front Plant Sci. 2015 Oct 28;6:905. doi: 10.3389/fpls.2015.00905 (PMC4623401; doi:10.3389/fpls.2015.00905)
Supplement: Supplementary file 2 [file Image2.PDF]

Supplementary Material

Nuclear proteome of developing wheat (*Triticum aestivum* L.) grain

Titouan Bonnot[1,2], Emmanuelle Bancel[1,2,*], Christophe Chambon[3], Julie Boudet[1,2], Gérard Branlard[1,2], and Pierre Martre[1,2,†]

*** Correspondence:** Emmanuelle Bancel: emmanuelle.bancel@clermont.inra.fr

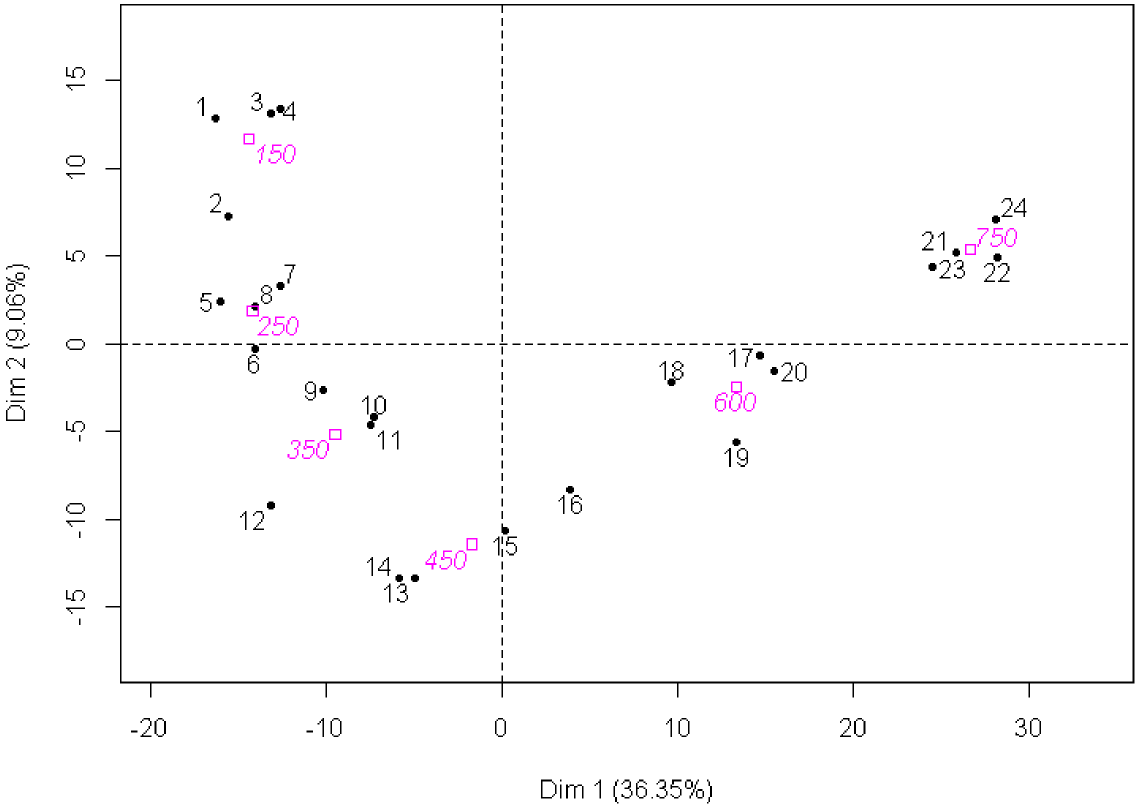

Supplementary Figure 2. Principal component analysis of protein spots detected by image analysis. Normalized volumes of protein spots detected in the four replicates were collected. 1-4: replicates at 150°Cd; 5-8: replicates at 250°Cd; 9-12: replicates at 350°Cd; 13-16: replicates at 450°Cd; 17-20: replicates at 600°Cd; 21-24: replicates at 750°Cd.
