# Supplementary material for: Changes in the nuclear proteome of developing wheat (Triticum aestivum L.) grain
Source: Front Plant Sci. 2015 Oct 28;6:905. doi: 10.3389/fpls.2015.00905 (PMC4623401; doi:10.3389/fpls.2015.00905)
Supplement: Supplementary file 3 [file Image3.PDF]

Supplementary Material

Nuclear proteome of developing wheat (*Triticum aestivum* L.) grain

Titouan Bonnot[1,2], Emmanuelle Bancel[1,2,*], Christophe Chambon[3], Julie Boudet[1,2], Gérard Branlard[1,2], and Pierre Martre[1,2,†]

*** Correspondence:** Emmanuelle Bancel: emmanuelle.bancel@clermont.inra.fr

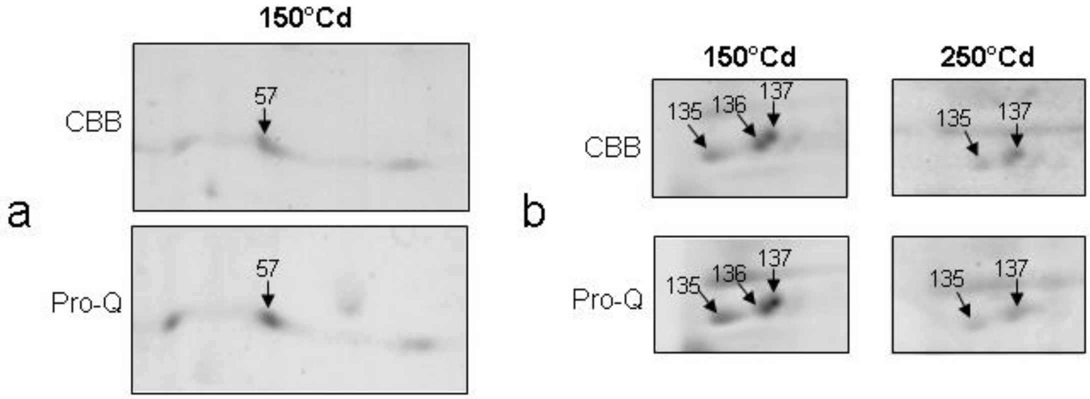

Supplementary Figure 3. Some nuclear protein spots phosphorylated during division / differentiation phase of wheat grain development. 2D gels were stained with Pro-Q Diamond (Pro-Q, Invitrogen) according to the manufacturer's instructions and adapted (Agrawal and Thelen, 2005) then stained with Coomassie Brilliant Blue G250 (CBB). Proteins spots stained both with Pro-Q and CBB were considered as phosphorylated. **a**: HMG1/2-like protein (spot 57) was phosphorylated at 150°Cd after anthesis. **b**: Histone deacetylase HDAC2 was phosphorylated at 150°Cd (spot 135, 136) and 250°Cd after anthesis (spot 135); Histone deacetylase HDT2 was phosphorylated at 150 and 250°Cd (spot 137) after anthesis.

Supplementary reference:

Agrawal, G. K., and Thelen, J. J. (2005). Development of a simplified, economical polyacrylamide gel staining protocol for phosphoproteins. *Proteomics* 5, 4684–8. doi:10.1002/pmic.200500021.
